# Neuropsychiatry

**Published:** 2014-08

**Authors:** Derryck H Smith

**Affiliations:** Vancouver, British Columbia

**Figure:**
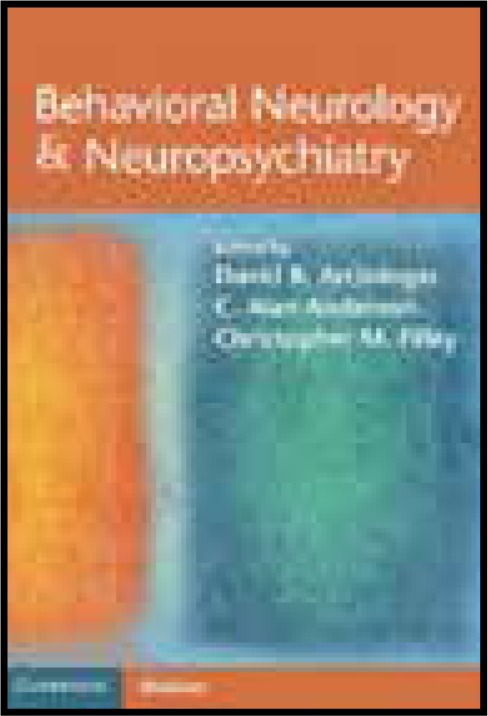


The purpose of this book is to exhaustively review what appears to be a general integration between the subspecialities of neurology and psychiatry. In the Foreward, it is pointed out that practitioners of neurology and psychiatry “have been assigned the uniquely challenging task of treating the most complex disorders of the most complex organ in the body.”^p xi^ The Preface notes that recent behavioural neurology and neuropsychiatry have merged into a single medical subspeciality, and this volume of more than 650 pages is a survey of the entire field. There are contributions from more than 50 different eminent experts in the field. This book is both exhaustive and comprehensive. One of the strengths of the book is that the 3 editors have made significant contributions to numerous different chapters. For example, Dr David B Arciniegas has written or coauthored 9 of the 38 chapters.

This book is well organized into a review of structural and functional neuroanatomy, followed by a second section on neurobehavioural and neuropsychiatric assessment, and a third section focusing on treatments and behavioural neurological and neuropsychiatry.

This volume has both the advantages and disadvantages of multiple authors in that there is a wide variety of expert opinion brought to bear, but also differences stylistically between the various chapters.

Many of the authors have published elsewhere. For example, Jonathan Silver and Thomas McAlister are 2 of the 3 coauthors for the second edition of the *Textbook of Traumatic Brain Injury*.^1^ Another well-published expert in the area of brain injury is James P Kelly.

Although the focus of this book claims to have a “carefully selected group of international experts,”^p xi^ in actual fact most of the authors are American. There are 2 authors from Australia and 1 from Spain, Israel, and Canada. The Canadian author is Morris Freedman from Baycrest Hospital and the University of Toronto.

This book is exhaustively referenced, which is a strength for readers seeking background information and more information on each of the chapters. There is a full section of colour plates illustrating brain anatomy and various imaging modalities.

This book is likely to become the standard textbook for programs offering subspeciality training in behavioural neurology and neuropsychiatry. I consider it to be an excellent volume and would strongly recommend it to psychiatrists with a subspeciality interest in the interface between neurology and psychiatry.
